# A Hybrid Generic Framework for Heart Problem Diagnosis Based on a Machine Learning Paradigm

**DOI:** 10.3390/s23031392

**Published:** 2023-01-26

**Authors:** Alaa Menshawi, Mohammad Mehedi Hassan, Nasser Allheeib, Giancarlo Fortino

**Affiliations:** 1 Information Systems Department, College of Computer and Information Science, King Saud University, Riyadh 11543, Saudi Arabia; 2 Department of Informatics, Modeling, Electronics, and Systems, University of Calabria, 87036 Rende, Italy

**Keywords:** UCI dataset, heart diseases, artificial intelligence (AI), machine learning (ML), deep learning (DL), feature selection, model voting, decision support system (DSS)

## Abstract

The early, valid prediction of heart problems would minimize life threats and save lives, while lack of prediction and false diagnosis can be fatal. Addressing a single dataset alone to build a machine learning model for the identification of heart problems is not practical because each country and hospital has its own data schema, structure, and quality. On this basis, a generic framework has been built for heart problem diagnosis. This framework is a hybrid framework that employs multiple machine learning and deep learning techniques and votes for the best outcome based on a novel voting technique with the intention to remove bias from the model. The framework contains two consequent layers. The first layer contains simultaneous machine learning models running over a given dataset. The second layer consolidates the outputs of the first layer and classifies them as a second classification layer based on novel voting techniques. Prior to the classification process, the framework selects the top features using a proposed feature selection framework. It starts by filtering the columns using multiple feature selection methods and considers the top common features selected. Results from the proposed framework, with 95.6% accuracy, show its superiority over the single machine learning model, classical stacking technique, and traditional voting technique. The main contribution of this work is to demonstrate how the prediction probabilities of multiple models can be exploited for the purpose of creating another layer for final output; this step neutralizes any model bias. Another experimental contribution is proving the complete pipeline’s ability to be retrained and used for other datasets collected using different measurements and with different distributions.

## 1. Introduction

The World Health Organization (WHO) declared that heart attacks are the main contributor to 31% of deaths worldwide [1]. In Gulf Cooperation Council countries, including the Kingdom of Saudi Arabia, the percentage is higher at 46% [2]. The Turkish Society of Cardiology, in a message issued on World Heart Day in 2015, stated that 300,000 heart attacks are observed every year in Turkey and 125,000 cases result in death [3]. Moreover, heart attacks causes death to more than a quarter of a million Americans [4]. Statistics show that heart disease alone costs over USD200 billion in the United States annually [4]. Additionally, according to the American Heart Association, health care costs for heart disease are estimated to double by 2030 [5].

Nausea, vomiting, and cold sweats can be symptoms that indicate a heart attack [1]. Fainting and loss of consciousness can be added to these parameters. For patients over a certain age, shortness of breath is also among the symptoms. These symptoms are experienced in approximately 80% of individuals who have had a heart attack. It occurs quietly in the other 20% without any previous symptoms. Eighty percent of cardiovascular diseases are developed owing to smoking, hypertension, genetic predisposition, obesity, sedentary lifestyle, and diabetes. Other causes are high cholesterol (low-density lipoprotein—sometimes called “bad” cholesterol—and triglycerides), (high-density lipoprotein —sometimes called “good” cholesterol), consumption of alcohol, presence of various heart diseases (such as vascular occlusion, arrhythmia, and experiencing a heart attack), and stress. However, the number of heart disease patients is expected to increase if precautions are not seriously considered [6]. Apart from adopting a healthy lifestyle, avoiding smoking, and following a healthy diet, accurate and timely diagnosis of heart disease with comprehensive analysis is another important factor that can play a role in saving patients’ lives [7]. 

The growing trend in digital health has introduced a growing opportunity for physicians to enhance patient diagnoses to be more accurate and decisive [1,8,9]. Recently, doctors have increased their dependency on digital technologies to enhance the decision-making process. In the business of health care, machine learning (ML) is transforming into being a key factor in helping the diagnosis of patients [10]. Generally, heart problems are remarkably critical, and they must be taken seriously. Regarding gender distribution, heart problems occur more frequently in males than females [11,12]. Therefore, in this paper we take a step toward saving the lives of heart disease patients by developing a data-driven generic framework to improve the diagnosis of patients based on their medical history. In this work, in-depth investigations have been undertaken to invent a novel prediction model that does not use distinct techniques but rather combines two or more techniques to come up with the best possible prediction output. These fused techniques are commonly known as hybrid methods [13]. Our main contribution is the development of a dynamic multi-model data-driven framework that is applicable to any heart disease dataset schema with an automated data pipeline and automated feature selection process based on a hybrid (not single) method with less-biased ML models.

The rest of this paper is organized as follows: in Section 2, we provide the literature review and analysis of current gaps in the research; in Section 3, we illustrate the scientific background and describe the ML and deep learning (DL) algorithms used in this work. In Section 4, we encapsulate the model details such as the dataset description, data pipeline, features selection framework, and classification framework. In Section 5, we outline the results and perform a comparative study of the existing research trials and the proposed work. Finally, in Section 6, we conclude this work and outline future research. 

## 2. Literature Review

The problem of heart disease detection has been addressed through multiple studies that combine multiple and different ML techniques [14,15,16]. Parthiban and Srivatsa [17] proposed a two-step solution: first, they used a support vector machine (SVM) to identify patients who already had diabetes. Second, they also exploited SVM for diagnosing heart disease. They accomplished 95% accuracy considering features such as blood sugar level, patient age, and blood pressure data. They were trying to employ an extremely common features set and did not carry out any special collection or handling to guarantee its applicability in any situation. Melillo et al. [10] built a CART-based ML model for automatic detection of congestive heart failure discriminating between high- and low-risk patients. The model sensitivity reached around 0.94, and specificity reached 0.64. Guidi et al. [18] proposed an early detection decision support system (DSS); the aim of this model is to detect heart problems early. The authors conducted a comparative analysis of multiple ML and deep neural network (DNN) models such as neural networks, SVM, and CART methods. The accuracy approached 88% and was achieved using random forest (RF); it outperformed any other classification model. 

Furthermore, Ismail et al. [19] applied the extreme learning machine algorithm, using a feedforward neural network on real heart disease data. The results showed that prediction performance was high, reaching 0.8 accuracy when predicting patients with heart disease. Miao et al. [20] developed an ensemble ML model that uses a heart disease dataset to predict disease existence among patients. The researchers applied an adaptive boosting algorithm to four different datasets separately, and the average accuracy was estimated to be 85.27%. However, the model suffered from overfitting that resulted from minimizing its training error and consequently incurred more testing errors than training errors in the testing phase. Dun et al. [21] exploited multiple ML and DL models for the diagnosis of the heart’s functional problems and ran hyperparameter tuning to enhance the accuracy. An artificial neural network (ANN) achieved high accuracy, exceeding 78% for the test data. In another study, Thomas and Princy [22] applied multiple ML and NN models to predict risk levels for heart disease patients. The results highlighted an increase in accuracy of risk-level prediction, which reached 80% accuracy when a greater number of attributes were used.

Deepika and Seema [23] proved that ML can be effective in predicting heart disease using a data-driven approach. Naïve Bayes, decision tree, SVM, and other models were applied to a Cleveland dataset, and the results indicated that SVM achieved the optimum results over the others. Xu et al. [24] used the same dataset to develop a risk prediction system using different ML techniques. The RF algorithm provided high accuracy in predicting the cardiovascular risk level with 91% accuracy and was therefore selected to be the basis classification strategy of the system. Gavhane et al. [25] applied a neural network approach using a multilayer perceptron to determine the potential risk of heart disease in patients based on historical data. Tabassian et al. [26], who investigated the effect of ML on diagnosing heart failure compared with other traditional medical approaches, recorded great improvements in accuracy when using ML techniques compared with other standard medical measurements. 

Mohan et al. [13] introduced a hybrid model to predict heart disease more accurately using the University of California Irvine (UCI) heart disease dataset as an experimental dataset. Their model combined RF with a linear model, and the resultant accuracy was 87.4%. RF was used to extract important features, then NN was used to predict the output class for the model. Alotaibi [7] tried to implement different ML techniques on a Cleveland dataset with the intention of improving the accuracy of heart failure prediction. The best results were gained from a decision tree algorithm with 93% accuracy. Furthermore, Shah et al. [27] applied different ML techniques to the Cleveland dataset to envision the probability of heart disease among patients. The results showed that K-nearest neighbor provided the highest accuracy. However, none of the researchers implemented a feature selection framework to dynamically select the features for the model; rather, they selected the features based on suggestions from different scholars in the literature. ML has also been applied in different medical contexts to improve the prediction of other diseases and has recorded a huge increase in accurate results [28,29].

A common situation in the ML field is the high dimensionality of the data (known as the “curse of dimensionality”). Not only is a large amount of memory and processing needed, but also the models tend to suffer from overfitting [12]. To reduce dataset dimensionality many models feature engineering/selection approaches that could be experimented with to eliminate data that do not have much importance in the dataset [30]. Previous researchers have proved that using feature selection and engineering as part of the model pipeline could remarkably enhance prediction accuracy. Ayatollahi et al. [31] built a model for diagnosing arrhythmia that focuses on heart rate variability. They proposed a multilayer perceptron (MLP) for performing the classification and achieved remarkable accuracy via features reduction using Gaussian discriminant analysis. Besides Gaussian discriminant analysis, principal component analysis has been exploited by multiple researchers as the initial choice for handling high-dimensional input. Rahhal et al. [32] proposed a DNN model for selecting the optimal set of features, consequently to be employed in heart failure diagnoses. Rajagopal and Ranganathan [33] set up a comparison of five different dimensionality reduction methods (unsupervised linear and unsupervised nonlinear). They used MLP as a classification layer for detecting cardiac arrhythmia. They achieved a 0.99 F1 score using Fast-ICA with 10 components as a minimum threshold. Singh et al. [34] explored the use of “generalized discriminant analysis” for the features extraction step (nonlinear features). Bashir et al. [35] applied different models of ML with the intention to improve prediction performance of heart disease using the feature selection approach. Naïve Bayes, RF, and other models were applied, and the results gained high accuracy measures with the feature selection approach. As discussed earlier, dimensionality reduction and feature engineering can enhance the quality of data explored and ultimately improve prediction accuracy [36]. 

After having performed the literature survey, we realized that a gap that still exists in the current body of knowledge, which needs to be filled. First, most of the research explored did not fulfill the multi-model approach and cannot be generalized to other datasets because the models were built specifically for the dataset under investigation with no attempt to investigate the model in other datasets for generalization purposes. Moreover, most of the results generated were built using a single model approach, so the result could be biased by this model, whereas adding an additional layer on top of a set of different models could overcome this problem and enhance the results from different models. The second pitfall is that some researchers selected features manually based on common features used in similar cases, which cannot be applied to other datasets automatically. This requires manual investigation of the dataset, which is time consuming and prone to human error. Other researchers have applied a single feature selection method to choose the features, whereas the hybrid approach that fuses the decisions of different methods and selects the best from among them could enhance the feature selection process and ensure more accurate results. 

The main idea behind this discussion is to stress the significance of ML approaches in enhancing the prediction accuracy of heart disease using a data-driven approach. In this research, the start point was a gap analysis of previous research trials. This gap analysis identified the contribution of this paper as follows:

- **Single schema dataset:**

Previous studies have been applied to a single dataset, and the models were developed and adopted to make predictions based on a single dataset schema with no applicability of working in different modes. In this work, the proposed system can operate in different modes, and it is neutral for input data quality because the data pipeline is completely automated. In addition, two datasets were exploited to ensure this privilege: the UCI dataset, which has been used for framework development and assessment, and another public dataset [19], which was used to validate the superiority of the proposed framework in neutralizing the dataset scheme effect.

- **Using a single feature selection mechanism:**

All previous researchers used a single feature selection approach for feature engineering. To the best of our knowledge, no previous work has been initiated to apply a multiple feature selection mechanism and select the best one according to the given dataset (the hybrid approach). In the proposed work, we built a hybrid voting-based model to select input features. The hybrid voting model employs multiple feature selection techniques and adds a voting layer. Consequently, the main contribution of this work is the ability to dynamically select the best features based on an input dataset.

- **Model bias:**

The current literature shows that using ML and DL algorithms can introduce bias into the results, which arises from the lack of fairness concept. The idea of fairness is based on data bias, such that the model learns the input data without realizing that there are missing data. The problem of data bias and model fairness has been tackled in the proposed model because it employs multiple ML and DL algorithms and builds a nonlinear additional voting layer to neutralize any model or data bias.

- **A proposed new Stacking Mechanism:**

The classical stacking architecture relies on multi-staging classifiers, and each layer output is input for the consequent layer. In the proposed framework, we propose and implement a new stacking classification architecture. The proposed stacking performs a similar task but adds the classifier decision probability to the classification decision and both become inputs for the consequent layer. The thinking behind this idea is to strengthen each classification result with probabilities so that they are considered in the consequent classification layer.

To address the gaps in the research, we attempt to improve the performance of heart disease prediction by developing a novel and generic framework that is agnostic to input data schema, and which includes a hybrid voting-based feature selection framework and hybrid multistage stacking classification framework that can be used for other problems. This research is not intended to replace the traditional medical approach used for diagnosing heart diseases; rather, we attempt to enhance this process with the invention of ML and DL techniques. 

## 3. Scientific Background

There are different types of algorithms used in this work, all of them belonging to the supervised learning category. The most commonly used ML algorithms in previous research were selected to be part of this study framework. ML approaches included in this research are: logistic regression (LR), SVM, RF, XGBoost, and DL. Every algorithm can outperform the others for a certain type and quality of data.

### 3.1. Logistic Regression (LR)

LR is one of the classical statistical approaches that, in its basic form, employs a “logistic function” to model a binary dependent variable. Mathematically, a binary logistic model has a dependent variable with two possible outcomes, in our case, such as: has heart problem/has not, which is defined by an indicator variable [37,38]. LR can be perceived as a “special-case” of the “generalized-linear model”; however, LR is based on different assumptions. The equation of LR is as follows [39]:

(1) $$Logit\left(p\right)=\ln\left(\frac{p}{1-p\;}\right)=\frac{prob.\;of\;presence\;of\;characteristics}{prob.\;of\;absence\;of\;charateristics}$$

### 3.2. Support Vector Machine (SVM)

SVM creates a hyperplane (or set of hyperplanes) in a high-dimensional space; these hyperplanes could be used for classification, regression, and removing outliers [40]. Fundamentally, a successful separation is accomplished by the hyperplanes that have the maximum distance from the closest training-data point of any class (functional margin). Apparently, achieving the maximum margin leads to obtaining the minimum generalization classification error of the classifier [41].

### 3.3. Random Forest (RF)

RF is an ensemble-learning-based algorithm for both classification and regression [41]. Regarding classification problems, the result from RF is the class picked by the major trees. Regarding regression problems, the mean of the individual trees is considered as an output. RF neutralizes the tendency of decision trees to overfit on the training data [42,43]. The three common methodologies used are: forest RI (random forest choice), forest RC (random blend), and a combination of forest RI and forest RC. 

### 3.4. XGBoost

Even taking into account ML competitions and Kaggle competitions, XGBoost is one of the top-performing algorithms that is picked initially for structured data [44]. XGBoost shows superiority in terms of speed, accuracy, and performance. It is referred to as the “enhanced gradient-boosting algorithm” that exploits the gradient-boosting framework in an efficient way. Boosting is another ensemble technique whereby previously mentioned model errors are tackled in new iterations [44]. 

### 3.5. Deep Learning (DL)

For the purpose of creating systems that acquire learning similar to how humans learn, the base architecture for DL was inspired by the base structure of a normal human brain. Consequently, some fundamental definitions within the scope of DL would be mapped to neurology [45]. Apparently, similarly to how neurons construct the primary basic blocks of the human brain, DL architecture includes “computational units” that permit the modeling of nonlinear functions (perceptrons) [46].

In this work, two models are exploited: DNN and CNN.

## 4. Proposed Framework

In this section, the framework components and operating flow are discussed in detail, starting with the dataset description and ending with the diagnosis decision. 

### 4.1. Dataset Description

The dataset used for this paper’s objective is a public dataset collected from the Kaggle platform, and its time interval starts from 1988. It is known as the UCI heart disease dataset [47] and includes information regarding clinical instances of heart disease with 76 columns and 75 features beside the label (prediction). It has four different databases contributed to by four different medical institutions: Cleveland Clinical Foundation, Long Beach Medical Center, the Hungarian Institute of Cardiology, and Switzerland University Hospital. All four datasets have the same instance format and within the 75 features, 13 fundamental features are commonly used in most previous research as per scholars’ suggestions. In our model, we have exploited these 13 features based on the platform recommendation, which is built to select the top influencing features, as will be described in the next section. A description of each attribute and its meaning is given in Table 1. 

### 4.2. Hybrid Feature Selection Framework

To verify the usage of only 13 features based on a data-driven selection process, a platform has been built to select the top influencing features. Some features have been dropped such as ID, name, and social security number; there is no need to model this exclusion step. The remaining attributes will provide the inputs for a feature selection framework. The framework is a data-driven selection process that explores the whole set of features and selects the top influencing features based on a novel voting process. The framework is a hybrid framework that exploits multiple and different feature selection methods at the same time and then votes for the best combination of features that gained the highest ranking among all methods. 

There are three basic types of feature selection step:

1. The filter method (chi-square has been exploited after numerical variables binning, Pearson correlation, and ANOVA coefficients);
2. Wrapper methods: these methods split the data subsets and use them to train a model; according to the model results, features are eliminated or added (a recursive features elimination method has been used in this research);
3. Intrinsic methods: data are split into different subsets and train the model and select the best subset based on model results (the Lasso regularization and decision tree methods were used).

**Figure 1:**
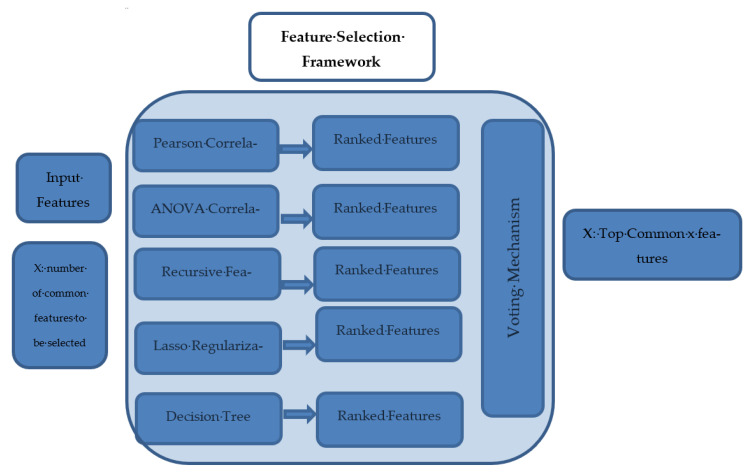
Feature selection framework.

The proposed framework can select the top features dynamically from a given dataset. To prevent any bias in the selection process, the model is designated as a hybrid model that exploits five different methods, run simultaneously, and produces a ranking score for each feature and a voting layer incorporated to vote for (x) top common features based on their ranking from each method. The framework at first accepts all datasets columns as input, and it takes the number of designated features (x) as a hyperparameter for the framework. In our framework, there are five different ML feature selection methods applied in parallel to the input dataset. Each method explores the whole set of features, measures the importance of each feature, and provides a set of top (x) important features (as requested by the user) that are ranked accordingly. Then, the next layer in the framework takes the ranking of each feature from the five different methods and votes on them to provide the top (x) common features according to their importance. In our case, 13 features were selected from the UCI dataset, and their importance was higher among the 75 features available. The 13 features derived from the framework are in compliance with the features recommended by the literature and used in previous research; however, we have selected them automatically based on the data-driven approach. The feature selection framework is shown in Figure 1. Moreover, Algorithm 1 is describing the feature selection pseudocode to clarify the steps in details.

**Algorithm 1.** Feature selection pseudocode1- Input: Data features set (S) and (x) is the designated number of selected features 2-Let M is the set of methods { pearson correlation, ANOVA, Recursive Elimination, Lasso and DT} 3-For every Algorithm (i) in (S) do the following:  3.1 Apply the following algorithms for measuring features importance  3.2 Rank the features importance for algorithm (i)  3.3 Save the features descending rank into global list (Fi) 4-for each Sorted list (j) in global list (Fi):  4.1 select the top (x) features and append in TOPi selected list 5-compute the intersection among TOP lists 6-Output: the intersection among the TOP lists

The features are ranked based on the score or coefficient values depending on the techniques used. For example, Pearson extracts the correlation coefficients, and DT extracts the features’ importance based on node impurity and so on. The next step is to outrank each feature within each method and identify the common top features to be used.

### 4.3. Data Preprocessing Pipeline

**Figure 2:**
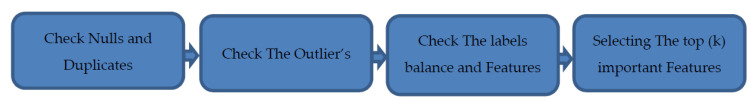
Data preprocessing pipeline.

In this work we proposed two different ways of building a model to diagnose heart diseases using a UCI dataset. The first approach was to skip all data preprocessing steps (outlier detection, distribution checking, skewness detection, etc.) and expose the dataset directly to the model. The second approach was to apply a complete data pipeline step to the data before exploiting any ML models for diagnosis. The data pipeline stages are illustrated in Figure 2.

#### 4.3.1. Check Nulls and Duplicates

The UCI dataset does not include null values but, for generalization purposes, a part of the pipeline has been built to check the nulls and impute them to consider any future dataset with null values inputted to the framework. Many imputing techniques are exploited, but the one employed in this research is the variable/label distribution and KNN imputation. The existence of duplicates in the dataset could affect model performance in two ways; first, it could introduce model bias for certain data points, and second, it might affect the model’s ability to generalize in case of one tuple in the training and the exact same duplicate existing in the testing set. In this work, one tuple was removed from the UCI dataset owing to duplication, and in general, any other dataset will be cleaned of such duplication.

#### 4.3.2. Check the Outliers

In this work, multiple outlier detection methods were used for tackling the issue of multi-variate outliers. Z-Score, Dbscan, and “isolation forest” (IF) were implemented and tested [12]. The experimental results confirmed our initial hypothesis that IF gives better results. Fundamentally, the IF technique is an isolation-based anomalies detection algorithm that constructs a binary tree to classify the data set. Further, the earlier the data item is labeled as a leaf, the greater the tendency to consider it as an outlier point. The algorithm steps start by randomly sampling multiple sample datasets and then construct many isolation trees based on classification of the field values. Then, for prediction, the algorithm compares an observation against a splitting value in a “node,” which will have two node children on which another random comparison will be done. The number of “splittings” made by the algorithm for an instance is named: “path length.” As expected, outliers would have shorter path lengths than the normal observations [12].

#### 4.3.3. Label Distribution

Label distribution inside the data set is a key factor in the model’s ability to generalize, and a balanced label distribution minimizes the model’s tendency to overfit. The UCI dataset label distribution illustrates that heart disease occurred in 54.46% of the dataset, and 45.54% had no heart disease. Although the distribution for the UCI dataset does not seem perfectly uniform, the variation can be neutralized by the model without any additional preprocessing steps. For a generic framework, we expect nonuniformity to exist more in real-world datasets; consequently, it is important to tackle this issue even if it does not appear in UCI clearly. In this work, oversampling and undersampling techniques are exploited to rebalance the labels. 

Another important step is examining the features individually to see if each one is normally distributed or not, and, if it is normally distributed, if it is skewed or not. The importance of this step appears in the modeling part such that all non-tree-based models are affected by features scales. Consequently, if the feature is normally distributed it will be standardized using the mean and standard deviation; however, if it is not, it will be scaled by its maximum value (after removing outliers). In the case of skewed normal distributed features, they will be transformed to non-skewed distribution using log-transformation.

### 4.4. The Proposed Classification Framework

**Figure 3:**
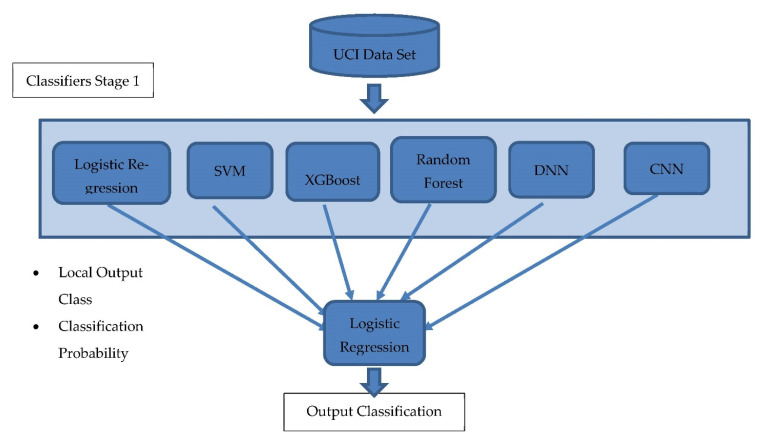
The proposed classification framework.

In this work, a hybrid multistage stacking classification model was proposed and implemented on a UCI dataset. The main contribution of this model is its ability to exploit multiple classification models simultaneously and avoid model bias in the case of results obtained from a single model only. Therefore, the model is considered hybrid in terms of having multiple classification models applied in parallel to the same dataset, and the results are evaluated by the next layer that selects the best among them. The results of each model (output class and classification probability) are introduced into a second-stage model, which is a binary classifier that votes for the final decision on whether the given patient has a form of heart disease. This proposed framework does not apply a simple voting technique, but it incorporates a novel voting technique that considers not only the counts of each class but also the probability of each class given from each classifier. This novel voting technique is considered to add value to the model for the following reasons: first, the second layer classifier has the ability to learn when each first layer classifier makes wrong or right classification decisions. This second layer learns from data how different classifiers react toward certain input and, based on the labels, it selects which classifiers should be fired for certain data feeds. Second, it can be more interpretable than simple voting because we can draw a boundary and extract knowledge of when each classifier can be decisive in the right way by considering the probability of each. Third, the overall model’s tendency to overfit decreases, and the model can be generalized for any dataset because it does not rely on a single model but on multiple models that are run in parallel, and the best results are considered among them. Finally, the idea of a second layer classifier decreases the overall model’s sensitivity whenever data feeds have quality issues. Worth noting is that this model differs from the traditional well-known stacking technique in terms of considering the decision probability as well as the decision itself as input for the second classification layer. The main advantage of this proposed model over stacking techniques is the enhanced accuracy of the second layer and the credibility of the decision in the first layer. This credibility is represented by the classification decision probability. Figure 3 shows the stages of the proposed classification framework with an indication of the specified output at each stage. 

**Figure 4:**
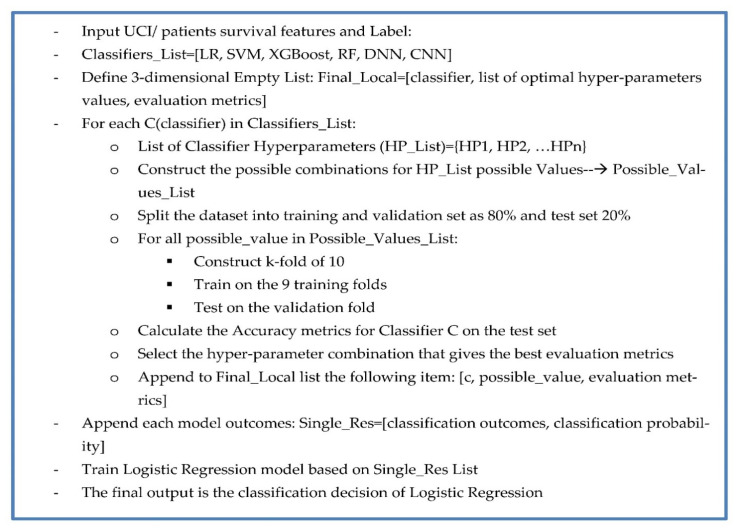
Framework steps.

The proposed model runs as the following pseudocode, Figure 4:

The first layer of classifier outcomes and prediction probabilities is passed to the second layer of classification (LR). This second layer is designed with the intention to reduce bias in the results and to try to generalize the results to add more predictive power and neutralize model bias as well as enabling stacking and voting capabilities. 

## 5. Experimental Results

In the evaluation step, there are many experiments and we included multiple evaluation metrics such as the confusion matrix, accuracy rate, precision, and recall. The confusion matrix is a table that embraces both the actual labels and predicted results. The results are defined in four basic components:

- True-positive (TP) values are true in both reality and prediction.
- False-positive (FP) values are false in reality but predicted as true.
- False-negative (FN) values are true in reality but predicted as false.
- True-negative (TN) values are false in both reality and prediction.

Let (N) be the number of all samples:

N = TP + FP + FN + TN(2)

N represents the total number of data points in the test set, and it consolidates all samples whether they have been predicted as correct or not.

The following metrics are commonly used for model evaluation (Table 2).

The experiments have been conducted for many design parameter variations: removing outliers and feature selection, running models separately, and multiple voting techniques. All the experiments divided the training and validation data into 10 folds (hyperparameter was selected among others). At every iteration, the k-fold partitioning was performed by keeping nine partitions for training and one for validation. The accuracy, sensitivity, and specificity of the results were measured to evaluate the performance of the model. Table 3 illustrates the average measurements over the 10 k-fold validation using ML and DL models separately on every design option either with preprocessing pipelines or without.

The optimal hyperparameters obtained for each model were calculated by grid search and the values are as follows:

- LR: Alpha = 0.1, Fit_intercept = true, Normalize = false, Solver = sag
- SVM: kernel = ‘rbf’, degree = 3, gamma = ‘scale’, coef0 = 0.0, shrinking = True
- XGBoost: verbosity =true, validate_parameters =false, min_split_loss = 0.001, max_depth =5, max_delta_step =0
- Random Forest: max_depth = 4, min_sample_split = 10, n_trees = 60, min_samples_leaf = 3
- DNN: hidden_layer_sizes = 3, activation = Relu, learning_rate = 0.01, solver = ’Adam’
- CNN: filter_size1 = 3, num_filters1 = 32, filter_size2 = 3, num_filters2 = 32, filter_size3 = 3, num_filters3 = 64, fc_size = 128, learning_rate = 0.01

The hyperparameters for some models were tuned using a grid search such as LR and RF, and others were tuned using a random search such as DNN and CNN owing to the huge number of hyperparameters and parameters to be tuned. 

Table 3 illustrates that CNN outperforms any other model; CNN employs three convolution layers followed by a dense layer and finally a softmax classifier. It can be interpreted from the results that CNN has the ability to handle nonlinear separable data via its dense layers and has the ability to neutralize the effect of biased data. In addition, multiple filter contents give it the advantage of allowing for generalization by extracting multiple data aspects. Another insight from the results is that applying data preprocessing pipeline steps (outlier detection, feature selection, etc.) can enhance the results whether the model is ML or DL. Clearly, some models are affected more than others, but the conclusion is that the data preprocessing pipeline can help to improve accuracy.

The proposed model was compared to another two models that embrace voting in different ways. The experimental results inductively prove the proposed model’s superiority over other voting mechanisms including classical stacking and traditional voting (where the voting layer counts the precedent classifiers’ outcomes and makes the final classification decision based on the majority of the decisions). Moreover, the proposed framework was compared with single-model CNN only; Table 4 portrays the results of each model and of the proposed model.

As illustrated in Table 4, the proposed model outperforms the traditional voting approach, classical stacking, and the single-model approach (CNN). For benchmarking purposes, a comparative study was conducted against other research trials that addressed heart problems and specifically worked on a UCI dataset (Table 5).

## 6. Framework Validation

One main purpose of this research was to design and develop a model that can be deployed and used in hospitals and clinics without rebuilding separate models for each entity according to its data and systems platforms. For the purpose of ensuring the ability of the proposed framework to be generalized and operable on any other dataset regardless of its contents of schema, the complete pipeline has been end-to-end tested and validated on a different dataset, namely, the Cardiovascular Heart Disease (CHD) dataset, which is available from the Public Library of Science [48]. The dataset contains records from 299 patients with heart failure collected in 2015. All 299 patients had left ventricular systolic dysfunction and had previous history of advanced stage of heart failures. The dataset contains 13 features, which report clinical, body, and lifestyle information. The dataset columns and a description are given in Table 6. 

For the CHD dataset, the proposed feature selection framework concludes that no features can be eliminated for the purpose of modeling and all features should be included with different importance and label correlation degrees. Regarding the preprocessing pipeline step, the CHD dataset has null values in multiple columns and needs to be resolved. The dataset has 60% survival and 40% death cases as labels; however, the distribution for this dataset does not seem perfectly uniform, but the variation can be neutralized by the model without any additional preprocessing steps. The classification stage was performed on the dataset with its features, and the results were recorded and compared with those of Chicco and Jurman [49], who have developed the dataset and made it publicly available for researchers. Chicco and Jurman [49] have explored different ML techniques in this dataset to predict patient survival. They have reached 0.83 accuracy in their work, as can be seen in Table 7.

As illustrated in Table 7, the proposed model gives better results than other work developed on the same dataset. The proposed framework has outperformed the other work with almost 9% higher accuracy.

## 7. Conclusions

In this paper, we proposed a new heart disease detection model that builds multiple models of prediction and uses the output probability distribution to produce a following prediction layer. The concept of considering first-layer classification output probability outperforms the normal voting or stacking technique because it minimizes model bias based on its output distribution. Another important aspect is ensuring pipeline reusability for different datasets with different columns and distributions. In this research we developed a generic framework for heart disease diagnosis that operates using multiple machine learning and deep learning techniques. The proposed multistage stacking framework contains two consequent layers. The first layer contains simultaneous machine learning models. The second layer consolidates the outputs of the first layer (classification results and probabilities) and classifies them as an advanced voting layer. The proposed model starts by filtering given dataset columns using multiple feature selection methods, and the selected features from that step continue for the rest of the pipeline. Results from the proposed system show superiority over using a single machine learning model or using traditional voting techniques or the classical stacking methodology. We concluded 96.3% accuracy over the testing set with a 10 k-folding partitioning structure. The proposed framework can operate in different modes, and it is neutral for input data quality because the data pipeline is completely automated. Moreover, it has built a voting-based model for input features prior to preprocessing, and a multistage classification step that employs multiple feature selection techniques and adds a voting layer that considers the ranked features and votes for the top common features. Consequently, the main impact of this study comes from demonstrating the ability to dynamically select the best features based on the input dataset. The model has been validated to ensure generalizability and has outperformed other models in terms of accuracy thus proving its outstanding contribution to heart diagnosis research.

## Figures and Tables

**Table 1:** The resultant features from the UCI dataset.

No.	Feature (Attribute Name)	Distinct Values
1	*Age*–Patient age (years)	Between 29 and 77
2	*Sex*–Gender of a patient	1 for males, 0 for females
3	*CP*–Level of chest pain a patient is suffering from when arriving at hospital (if exists)	0, 1, 2, 3
4	*Chol*–The cholesterol level recorded when patient is admitted to hospital	Between 126 and 564 (mg/dL)
5	*RestBP*–The blood pressure (BP) figure for the patient at the time of admission to hospital	Between 94 and 564 (mm Hg)
6	*FBS*–The fasting blood sugar of the patient with binary classification: if more than 120 mg/dL =1 else =0	0, 1
7	*RestECG*–The result of resting electrocardiographic (ECG) from 0 to 2, where each value describes the severity of the pain	0, 1, 2
8	*HeartBeat*–The maximum value of heartbeat counted when patient is admitted	Between 71 and 202
9	*Exang*–Used to understand whether exercise induced angina or not. Yes = 1 and not = 0	0, 1
10	*Oldpeak*–Defines the patient’s depression status	Real numbers between 0 and 6.2
11	*Slope*–Patient’s condition during peak exercise. The value is defined by three segments [Up sloping, Flat, Down sloping]	1, 2, 3
12	*Ca*–The status of fluoroscopy. It shows how many vessels are colored	0, 1, 2, 3
13	*Thal*–A kind of test required for patients with chest pain or breathing difficulty. Four different values showing the result of Thallium test	0, 1, 2, 3
14 (Class)	*Target*–The class or label column. There are two types of classes (0, 1), where “0” indicates that the patient has no heart disease, whereas “1” implies that the patient has heart disease based on the features used in the modeling process.	0, 1

**Table 2:** Evaluation metrics.

Metric	Definition	Formula
Accuracy	The overall truly predicted samples divided by overall samples	(TP + TN)/N
Specificity	The percentage of actual negative samples that were predicted as negative	TN/(FP + TN)
Sensitivity (Recall)	The percentage of actual positive samples that were predicted as positive	TP/(FN + TP)
Precision	How many of the positively classified samples were actually positive	TP/(TP + FP)
F1 Score	The harmonic means of both recall and precision	2(recall * precision)/(recall + precision)

**Table 3:** Results for separate models (UCI dataset).

Without Preprocessing Pipelines				With Preprocessing Pipelines			
Model	Accuracy %	Specificity	Sensitivity	Model	Accuracy %	Specificity	Sensitivity
LR	82.1	79.8	85.6	LR	84	82.5	85.6
SVM	83.3	80	78.5	SVM	84.6	82.6	83.5
XGB	85.7	83.4	81.8	XGB	88.1	85.0	83.9
RF	81.4	80.2	79.3	RF	82.8	80.5	80.4
DNN	83.9	80.9	80.4	DNN	85.4	83.1	82.2
CNN	91.2	87.2	84.9	CNN	93.3	88.0	86.1

**Table 4:** Results for the proposed framework on UCI.

With Removing the Outliers and Feature Selection			
Model	Accuracy %	Specificity	Sensitivity
Proposed Framework on HDD	96.3	91.9	93.1
Traditional Voting Framework	92.5	91.0	89.3
Classical Stacking	93.9	91.7	92.4
CNN	93.3	88.0	86.1

**Table 5:** Comparative study.

Authors	Methodology and Results
Shah et al. [27] (2020)	Accuracy around 90.7% Implemented multiple classification techniques. Decision Tree, KNN and K-Means were compared. Concluded that accuracy obtained by KNN was highest. Selected features based on literature surveys.
Alotaibi [7] (2019)	Accuracy around 93% Compared multiple ML algorithms. The accuracy of Decision Tree, Logistic Regression, Random Forest, Naive Bayes and SVM classification algorithms were compared. Decision tree algorithm had the highest accuracy. Selected features based on literature surveys.
Mohan et al. [13] (2019)	Accuracy 87.4% Hybrid model combining RF and LM. RF used to extract features and then NN was used to predict the results and the hybrid model was compared to other ML techniques. Their hybrid model showed 1% improvement compared to the other techniques. 13 features extracted by RF and used in the model.
Deepika and Seema [23] (2017)	Accuracy around 95% Compared multiple ML algorithms Naïve Bayes, Decision tree, SVM and ANN methods were compared. SVM gained the optimum results. Selected features based on literature surveys.
Shu et al. [24] (2017)	Accuracy around 91% Compared multiple ML algorithms Random Forest, C4.5, SVM, Bayes, RBF network, AdaBoost were compared. Random Forest provided the best accuracy. Includes features selection framework but not hybrid model.
Mioa et al. [20] (2016)	Average accuracy around 85% Used advanced integrated ML (Adaptive boosting algorithm) Applied on 4 different datasets of UCI Used 29 features Suffered from overfitting
Proposed Model	Accuracy reached 96.3% Hybrid multi-stage stacking classification framework that can be generalized for other problems. Includes hybrid feature selection framework. Framework is agnostic to input data schema.

**Table 6:** CHD dataset.

No.	Feature (Attribute Name)	Measure	Distinct Values
1	*Age*–Patient age (years)	Years	Between 40 and 95
2	*Sex*–Gender of patient	Boolean	1 (male) 0 (female)
3	*Anemia*–Decrease of red blood cells or hemoglobin	Boolean	0, 1
4	*High blood pressure*–If a patient has hypertension	Boolean	0, 1
5	*Creatinine phosphokinase (CPK)*–Level of the CPK enzyme in the blood	Mcg/L	[23, …, 7861]
6	*Diabetes*–If the patient has diabetes	Boolean	0, 1
7	*Ejection fraction*–Percentage of blood leaving the heart at each contraction	Percentage	[14, …, 80]
8	*Platelets*–Platelets in the blood	kiloplatelets/mL	[25.01, …, 850.00]
9	*Serum creatinine*–Level of creatinine in the blood	mg/dL [0.50, …, 9.40]	mg/dL [0.50, …, 9.40]
10	*Serum sodium*–Level of sodium in the blood	mEq/L	[114, …, 148]
11	*Smoking*–If the patient smokes	Boolean	0, 1
12	*Time*–Follow-up period	Days	[4, …, 285]
13 (Class)	*Death event (Target)*–If the patient died during the follow-up period	Boolean	0, 1

**Table 7:** Results for the proposed framework on CHD dataset.

Model	Accuracy %
Proposed Framework on CHD Dataset	91.8
Chicco and Jurman [49]	83

## Data Availability

Heart Disease Cleveland UCI can be found at the following link: https://www.kaggle.com/datasets/cherngs/heart-disease-cleveland-uci (accessed on 15 December 2021), Cardiovascular Heart Disease (CHD) dataset can be found at the following link: https://plos.figshare.com/articles/dataset/Survival_analysis_of_heart_failure_patients_A_case_study/5227684/1 (accessed on 2 April 2022).
